# Strategies to Reverse Endothelial Progenitor Cell Dysfunction in Diabetes

**DOI:** 10.1155/2012/471823

**Published:** 2012-02-22

**Authors:** Alessandra Petrelli, Raffaele Di Fenza, Michele Carvello, Francesca Gatti, Antonio Secchi, Paolo Fiorina

**Affiliations:** ^1^Transplantation Research Center (TRC), Nephrology Division, Children's Hospital and Brigham and Women's Hospital, Harvard Medical School, Boston, MA 02115, USA; ^2^Transplantation Medicine, San Raffaele Scientific Institute, 20132 Milan, Italy; ^3^General Surgery, San Raffaele Scientific Institute, 20132 Milan, Italy; ^4^Transplantation Medicine, Università Vita-Salute San Raffaele, 20132 Milan, Italy

## Abstract

Bone-marrow-derived cells-mediated postnatal vasculogenesis has been reported as the main responsible for the regulation of vascular homeostasis in adults. Since their discovery, endothelial progenitor cells have been depicted as mediators of postnatal vasculogenesis for their peculiar phenotype (partially staminal and partially endothelial), their ability to differentiate in endothelial cell line and to be incorporated into the vessels wall during ischemia/damage. Diabetes mellitus, a condition characterized by cardiovascular disease, nephropathy, and micro- and macroangiopathy, showed a dysfunction of endothelial progenitor cells. Herein, we review the mechanisms involved in diabetes-related dysfunction of endothelial progenitor cells, highlighting how hyperglycemia affects the different steps of endothelial progenitor cells lifetime (i.e., bone marrow mobilization, trafficking into the bloodstream, differentiation in endothelial cells, and homing in damaged tissues/organs). Finally, we review preclinical and clinical strategies that aim to revert diabetes-induced dysfunction of endothelial progenitor cells as a means of finding new strategies to prevent diabetic complications.

## 1. Introduction

Endothelial progenitor cells (EPCs) are a subset of bone-marrow-derived cells committed to the maintenance and preservation of vascular turnover, remodeling, and homeostasis [1]. EPCs are immature cells, endowed with the capacity to be mobilized from the bone marrow into the bloodstream in response to growth factors and cytokines release [2, 3]. EPCs may differentiate into endothelial cells and finally take part in the vascular repair [2, 3]. Since 1997, when Asahara et al. published on Science the discovery of a population of circulating CD34^+^ cells showing proliferative capacity and ability to differentiate into mature endothelial cells *in vivo *and *in vitro *[4], much debate on EPCs origin and controversies on the appropriate isolation method was generated and several acronyms have been used to refer to this cell population. Different markers have been used to describe i*n vivo* circulating EPCs, among them we should mention CD34^+^VEGFR2^+^, CD34^+^CD133^+^VEGFR2^+^, CD133^+^VEGFR2^+^, and CD133^+^VeCadherin^+^ [5]. Several *in vitro* culture methods to isolate EPCs have been reported as well: colony-forming unit-endothelial cells (CFU-ECs), circulating angiogenic cells (CACs), and endothelial colony forming cells (ECFCs) [6]. Early EPCs exhibit a spindle-shaped morphology *in vitro*, have poor proliferative capacity, and produce to high extent angiogenic cytokines (e.g.; VEGF), while late EPCs show a cobblestone morphology *in vitro*, highly proliferative activity, and the ability to directly incorporate into capillary vessels [6]. However, despite the several subtypes of EPCs, there is agreement in the literature that EPCs from healthy subjects are able to repair blood vessels wall and that dysfunctional EPCs are defective in angiogenic properties, thus contributing to vascular diseases and progression of cardiovascular syndrome [7]. Thus, healthy EPCs might represent a precondition for a functioning cardiovascular system. Indeed, EPCs number and function have been reported to be impaired in type 1 (T1D) and type 2 (T2D) diabetes [8, 9] as well as in presence of cardiovascular risk factors [10–12], while a normalization of EPC function was found in euglycemic islet-transplanted patients [13], despite immunosuppressive treatment, thus justifying the improvement of diabetic complications in these patients. We will review how diabetes interferes with EPC function and subsequently summarize potential strategies to restore/repair EPC function in diabetic patients.

## 2. EPC Dysfunction in Diabetes

Diabetes and hyperglycemia may affect EPC function at each step of their lifetime. In this section we provide evidence of the current knowledge on diabetes-induced damage during EPC lifespan.

### 2.1. Mobilization from Bone Marrow

Several studies have focused on diabetes-mediated impaired EPC recruitment in the peripheral blood. Hyperglycemia was shown to affect bone-marrow-harbored EPCs by generating a diffused endothelial damage, microvascular remodeling, and reduction in c-kit^+^ Sca-1^+^ cells [14] in chemically induced (STZ) diabetic mice. Moreover, in this model, EPC deficiency was associated with an increased oxidative stress, DNA damage, and cell apoptosis [14]. The molecules involved in EPC mobilization process from bone marrow are circulating molecules like SDF-1*α* [15, 16], VEGF [17], GM-CSF [18], IL-8 [19], and cleaving enzymes [2]. SDF-1, which interacts with CXCR-4 receptor on target cells, is released by ischemic tissues [15] (via a HIF-1*α*-mediated induction) and is involved in EPC mobilization [15], homing into vascular structures [15], and differentiation [16]. Similarly, the role of VEGF in EPC mobilization has been widely studied in both humans and mice showing that, following acute ischemic injury, plasma levels of VEGF increase rapidly leading to a 50-fold increase in EPC percentage in the peripheral blood [17]. Among the several mechanisms involved in the impaired bone marrow mobilization of EPCs in diabetes, endothelial nitric oxide synthase (eNOS) dysfunction has been clearly demonstrated [20, 21]. Since uncoupling of eNOS leads to superoxide anion formation instead of nitric oxide (NO), Thum et al. hypothesized that such an altered enzyme activity could have a role in the reduction of EPC number in diabetic patients because of hyperglycemia-mediated increased oxidative stress [20]. Moreover, in streptozotocin-induced diabetic rats, EPCs were 39% less than in controls and this was associated with eNOS uncoupling in the bone marrow [20]. In a model of hind-limb ischemia-reperfusion (I/R) injury, plasma levels of VEGF and SDF-1*α* were measured and EPCs mobilization after ischemic injury was studied in diabetic rats and compared to euglycemic rats [22]. In this study, diabetic rats proved to be unable to mobilize EPCs after ischemic injury and this evidence was associated with a reduced release of VEGF and SDF-1*α* from ischemic muscle [22]. Interestingly, Gallagher et al. confirmed the relationship between SDF-1*α* reduced production and impaired EPCs peripheral counts in a diabetic murine model of wound healing [21]. Beyond soluble molecules, cleaving enzymes have shown a relevant role in EPCs mobilization: cathepsins (in particular Cathepsin L was shown to be essential for autoimmune diabetes in mice [23]) and elastases are released by neutrophils under conditioning with G-CSF and promote the cleavage of bonds between cells and stroma and the cleavage of SDF-1*α*/CXCR-4 interaction, thus inducing EPCs shedding; finally, MMP-9, a proteolytic enzyme found to be activated in diabetes [24], is essential for VEGF and SDF-1*α*-mediated EPCs mobilization [2]; indeed eNOS knockout mice (which mediates VEGF and SDF-1*α* signaling) promotes a reduced MMP-9 activity and an impaired MMP-9-mediated progenitor cells release [25, 26].

### 2.2. Trafficking

Once EPCs have been mobilized in the bloodstream, they migrate to the sites of ischemia/damage, in a process known to be mediated by SDF-1*α* [15] and VEGF [27]. Segal et al. demonstrated that EPCs harvested from patients affected by T1D and T2D in presence of SDF-1*α* showed an impaired migration compared to healthy control subjects [28]. The isolated EPCs were also characterized by a reduced cytoskeleton plasticity [28]. Interestingly, they demonstrated that treatment with exogenous NO corrects both migration defect and deformability impairment of diabetic EPCs [28]. Moreover, glucose-dependent and protein kinase C- (PKC-) mediated eNOS uncoupling, which results in hyperproduction of ROS rather than NO production, is associated with defective migratory capacity of EPCs from diabetic patients compared to nondiabetic controls [20]. Leicht et al. observed that late EPCs isolated from patients with T2D had impaired proliferation and migratory capacity compared to cells isolated from young healthy donors or non-diabetic age-matched subjects [29]. Advanced glycation end-products (AGEs) are known to accumulate in diabetes and were proven to impair migration and enhance apoptosis in EPCs cultured from human umbilical cord blood [30]. These effects were inhibited by anti-RAGE antibodies [30]. These data were confirmed by Sun et al. that challenged EPCs with AGE-human serum albumin at different concentrations and found that it significantly decreased EPCs migration [31]. Another way in which diabetes may alter EPCs' trafficking is lipotoxicity. It is known that oxidized LDL (Ox-LDL) is associated with reduced number and increased senescence of EPCs and these effects seem to be related to Akt activation, p21 expression, and p53 accumulation [32].

### 2.3. Survival

EPC trafficking in the bloodstream are more susceptible to diabetes-induced apoptosis. Indeed, a lower EPCs peripheral count has been described in diabetic murine models [21, 33]. Nevertheless, several studies have associated diabetes with reduced EPCs number when cultured *ex vivo*, due to both an increased apoptosis [30, 31, 34] or diminished proliferation [29, 32, 35, 36]. In our study, the percentage of circulating EPCs did not differ between T1D patients, islet-transplanted insulin-independent patients, and healthy controls, and no significant differences in apoptosis could be found among these subjects [13]. However, *in vitro* studies showed reduced number and increased apoptosis of diabetic-derived EPCs while a normalization of both parameters was evident in islet-transplanted patients. Lower secreting levels of IL-8 from EPCs cultured from T1D patients and a dose-dependent decrease of control EPCs number in presence of IL-8 antagonist (anti-IL-8) induced to speculate on the role of this chemokine in angiogenesis [13]. Several other investigators reported a reduced survival of EPCs cultured *ex vivo *in hyperglycemic conditions. Chen et al. cultured different subtypes (early and late) of EPCs with high glucose demonstrating a dose-dependent reduction of early EPCs number, reduced proliferation, and impaired migration ability of late EPCs compared to mannitol treatment [36]. High-glucose-mediated negative effects were restored by NO treatment and worsened by PI3K or eNOS inhibition [36]. Interestingly, it has been recently shown that treatment with adiponectin of human and murine EPCs prevents accumulation of high-glucose-induced premature senescence [33]. Other intracellular pathways have been demonstrated to be involved in EPCs survival in diabetes. The p38 MAPK pathway is activated in EPCs exposed to high glucose, inducing a dose-dependent reduction of *ex vivo* cell counts [35]. Finally, renin-angiotensin-aldosterone system has been described to be involved in EPCs survival process. Indeed, angiotensin II was shown to induce EPCs senescence [37] and aldosterone to downregulate VEGFR-2 expression leading to reduced EPCs number *ex vivo* [38]. These findings acquire interest considering that diabetes correlates with significantly higher circulating levels of angiotensin II and aldosterone [39] and that ACE inhibitors are available in clinical practice.

### 2.4. Homing and Differentiation

Investigators have outlined several crucial pathways involved in EPCs homing and differentiation. Interaction between SDF-1*α* and CXCR-4 is again fundamental, given that blockade of either the ligand or the receptor prevents recruitment to injured sites [15]. In 2007, Gallagher et al. demonstrated that the mechanism involved in diabetes-mediated EPCs dysfunctional homing is a reduced local release of SDF-1*α* and NO in the sites of wound and that SDF-1*α* exogenous administration could lead to a faster recovery of the wound [21]. Impaired capacity of EPCs to support endothelial tube formation was evidenced in T1D patients as well [8]. Marchetti et al. determined the effects of glucotoxicity on EPCs in *de novo* tube formation by culturing isolated EPCs from healthy donors with high glucose or high glucose plus benfotiamine, a scavenger of glucotoxicity [40]. While glucotoxicity led to impaired EPCs-mediated tube formation on matrigel (associated with a reduced activity of FoxO1) [40], benfotiamine could restore both FoxO1 activity and EPCs differentiation [40]. Another study showed that chronic incubation of EPCs isolated from healthy donors with high glucose levels impaired tube formation capability *in vitro* (decreasing eNOS and NO availability) [36], but could be improved by coincubation with NO [36]. Finally, the same mechanisms involved in EPC trafficking dysfunction are also relevant in homing and differentiation process.

## 3. Preclinical Experience in Reverting Diabetes-Mediated EPCs Damage

Several successful approaches to revert diabetes-induced EPCs dysfunction have been described in preclinical models. Herein, they are listed according to whether they have been performed *in vitro* or in animal models. 

### 3.1. In Vitro Studies

**Antioxidants**

Antioxidants are relevant mediators of EPCs impairment. Indeed, Ceradini et al. demonstrated that glyoxavlase 1 overexpression, an antioxidant key factor that modifies HIF-1*α*, restored high glucose-induced impairment of CXCR-4 and eNOS expression in EPCs [41]. Moreover, glucose-induced impairment of human EPCs was shown to be reverted by benfotiamine administration which modulates the PI3K/Akt/FoxO1 pathway [40]. Adiponectin-based conditioning of EPCs isolated from both human peripheral blood or mouse bone marrow prevented high glucose-induced senescence that was characterized by decreased ROS accumulation [33].

**Antidiabetic Drugs**

Currently used antidiabetic drugs showed beneficial effects on EPCs number, and function. Liang et al. cultured EPCs from healthy donors with AGEs and rosiglitazone [34]. Indeed, rosiglitazone was able to reduce EPCs apoptosis, to increase cell number and to enhance migration capacity [34]. Interestingly, insulin was shown to increase angiogenic potential of EPCs via IGF-1 receptor signal in both healthy donors and T2D patients [42].

**Gene Therapy**

Several approaches aiming to restore EPCs function by knocking down or overexpressing target genes were tested in mice models. Di Stefano et al. showed that EPCs harvested from p66ShcA knockout mice were resistant to high glucose injury [43]. Diabetic EPCs in which p53 gene was deleted did not exhibit senescence and form regular vascular-like structures [32]. Finally, *ex vivo* VEGF gene transfer in EPCs enhanced EPC proliferation, adhesion, and incorporation into endothelial cell monolayers [44].

### 3.2. Animal Studies

**Bone Marrow Mobilizing Factors**

In 1999 Takahashi et al. observed that GM-CSF increased circulating EPCs in rabbits and caused an improvement in hindlimb vascularization [18]. In a model of hindlimb ischemia-reperfusion, it was shown that preconditioning with G-CSF and SDF-1*α* could partially recover impaired postischemic progenitor cell mobilization in diabetic rats [22]. Moreover, Gallagher et al. showed that administration of SDF-1*α* into wounds of diabetic mice reverted EPC altered homing [21]. Our group recently showed that the targeting of the CXCR4-SDF-1*α* axis in diabetic mice induced an increased release and engraftment of endogenous EPCs leading to neoangiogenesis and improved ability to heal diabetic wounds [45].

**Cell Therapy**

Tamarat et al. administered bone marrow mononuclear cells from either non-diabetic or STZ-induced diabetic mice into a mouse model of hindlimb ischemia, which in turn was either diabetic or non-diabetic [46]. Administration of diabetic bone-marrow-derived cells to non-diabetic mice improved neovascularization (compared to saline infusion) in a less extent than the infusion of non-diabetic cells, while injection of non-diabetic bone-marrow-derived cells into diabetic mice improved blood flow recovery, capillary number, and ischemic/non-ischemic angiogenic score compared to the infusion of diabetic bone-marrow-derived cells [46].

**Drugs for Cardiomethabolic Control**

PPAR-*γ* agonists were demonstrated to increase mobilization of bone-marrow-derived progenitor cells via stimulation of Akt pathway [47]. ACE or HMG-CoA reductase inhibition resulted in significant increases of EPCs levels [48]. Moreover, ACE inhibitors proved to increase bone marrow ERK phosphorylation and MMP-9 activity, while statin-based therapy led to enhancement of bone marrow VEGF levels, Akt phosphorylation, eNOS activity, and normalized ROS levels [48]. EPCs peripheral levels, during the early postmyocardial ischemia phase, were increased by ACE inhibitors or statins treatment in rats, and this effect was also associated with improved cardiac function and enhanced capillary density in the peri-ischemic area [48]. Enalapril-treated mice showed a significant enhancement in circulating progenitor cell levels and a sixfold increase in bone marrow contribution to neoangiogenesis [49]. Interestingly, recent data showed that insulin resistant rats showed an insulin-signaling defect in EPCs that reduces EPC survival and that can be reversed by knocking down NF-kB [50] (see next paragraph).

**Gene Therapy**

Prevention from diabetes-mediated impairment of *in vivo* angiogenesis has been shown in p66ShcA knockout mice [43]. A recent study by Desouza et al. showed that infusion of EPCs, which were knocked down for NF-kB, led to a decrease in neointimal hyperplasia after carotid angioplasty in a model of type 2 diabetes [51]. Recently, a Phase I clinical trial showed an increase in neoangiogenesis after intramuscular gene transfer of plasmid encoding human VEGF in patients with critical limb ischemia [52].

**Restoration of Insulin-Producing Beta Cells Function**

We have recently shown that restoration of normoglycemia by successful islet transplantation induced increased number and improved angiogenic ability of EPCs compared to T1D [13].

## 4. Clinical Experience and Perspectives in Reverting Diabetes-Mediated EPCs Damage

### 4.1. Improvement of Glycometabolic Control

Optimized glucose control is undoubtedly associated with a better outcome of macro- and microvascular complications in patients affected by diabetes [53]. We demonstrated that insulin-independent islet-transplanted patients showed a recovery of EPCs number and function [13]. Interestingly, diabetes-mediated EPCs dysfunction has been demonstrated to be reversed in obese (non-diabetic) subjects after weight loss [54] meaning that the damage does not seem to be irreversible.

### 4.2. ACE Inhibitors

Routinely administered drugs as ACE inhibitors and angiotensin receptor blockers proved to benefit EPCs function [55, 56], even though no randomized clinical trials are available yet, thus suggesting to capitalize on this secondary effect and improve vascular function in diabetic patients. Bahlmann et al. investigated the effects of angiotensin II-receptor blockers, olmesartan and irbesartan, on EPCs in patients with T2D [56]. In both cases, ACE inhibitors increased peripheral number of EPCs compared to placebo treatment [56]. In patients with coronary artery disease, treatment with ACE-inhibitor ramipril was associated with increase in both peripheral cell count, and functional activity of EPCs, the latter being assessed by proliferation, migration, adhesion and formation of vascular structures *in vitro *[55].

### 4.3. Ex Vivo Conditioning

**Figure 1:**
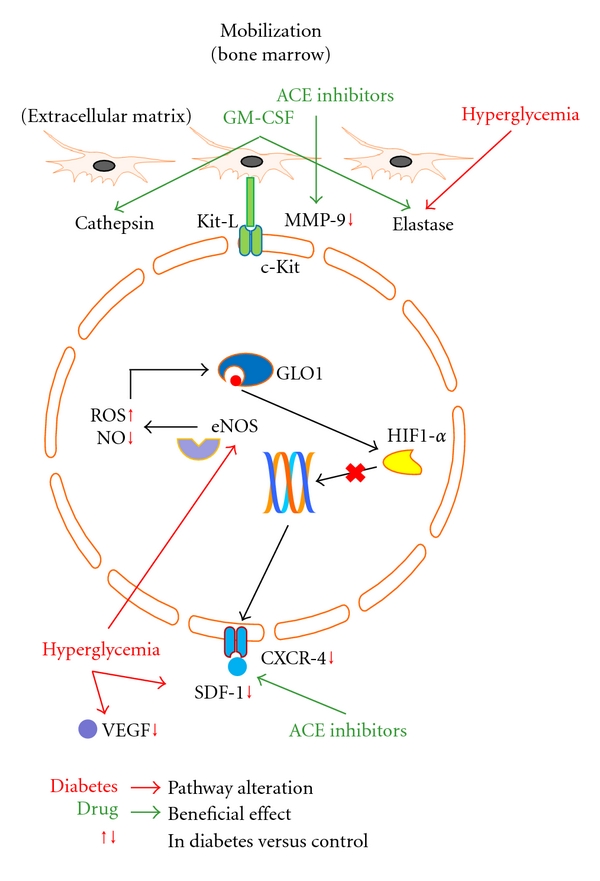
Pathways involved in diabetes-induced EPCs toxicity and possible strategies to reverse EPCs damage during bone marrow mobilization. EPCs recruited from bone marrow are here represented. Hyperglycemia alters CXCR-4/SDF-1*α* pathway, reduces VEGF levels, increases eNOS-mediated production of ROS and a reduction in cleaving enzymes activity. ACE inhibitors and GM-CSF administration improve bone marrow ability to shed EPCs in the periphery. Diabetes-specific metabolic alterations are in red, linked by red arrows to the pathways they interfere with. Red vertical arrows, next to intracellular or extracellular molecules, indicate that their concentration is diminished or increased in diabetic condition compared to nondiabetic status. Drugs with beneficial effect on EPCs are in green, linked by green arrows to the pathways they interact with. *MMP-9: matrix metalloproteinase-9; GLO-1: glyoxalase-1; ROS: reactive oxygen species; NO: nitric oxide; eNOS: endothelial nitric oxide synthase; HIF1-*α*: hypoxia inducible factor 1-*α*; SDF-1*α*: stem cell-derived factor-1*α*; CXCR-4: C-X-C chemokine receptor type 4; VEGF: vascular endothelial growth factor; Kit-L: c-Kit ligand. *

**Figure 2:**
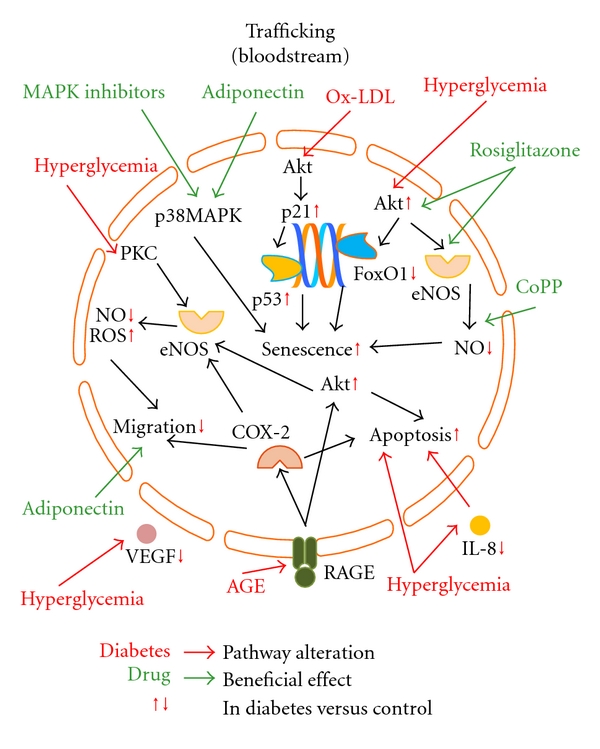
Pathways involved in diabetes-induced EPCs toxicity and possible strategies to reverse EPCs damage during trafficking in the peripheral blood. EPCs trafficking in the peripheral blood are here represented. Hyperglycemia, Ox-LDL, and AGEs accumulation induce an impaired migration ability and reduced cell counts by either increased senescence or increased apoptosis of EPCs in both *in vivo* and *in vitro *assays. Statins, Adiponectin, CoPP, and MAPK inhibitors are able to reverse diabetes-mediated damage on circulating EPCs. Diabetes-specific metabolic alterations are in red, linked by red arrows to the pathways they interfere with. Red vertical arrows, next to intracellular or extracellular molecules, indicate that their concentration is diminished or increased in diabetic condition compared to nondiabetic status. Drugs with beneficial effect on EPCs are in green, linked by green arrows to the pathways they interact with. *ROS: reactive oxygen species; NO: nitric oxide; eNOS: endothelial nitric oxide synthase; VEGF: vascular endothelial growth factor; Ox-LDL: oxidized low-density lipoprotein; MAPK: mitogen-activated protein kinase; CoPP: cobalt protoporphyrin; AGEs: advanced glycation end-products; RAGE: receptor for AGE; PKC: protein kinase C; IL-8: interleukin-8; COX-2: cyclooxygenase-2. *

**Figure 3:**
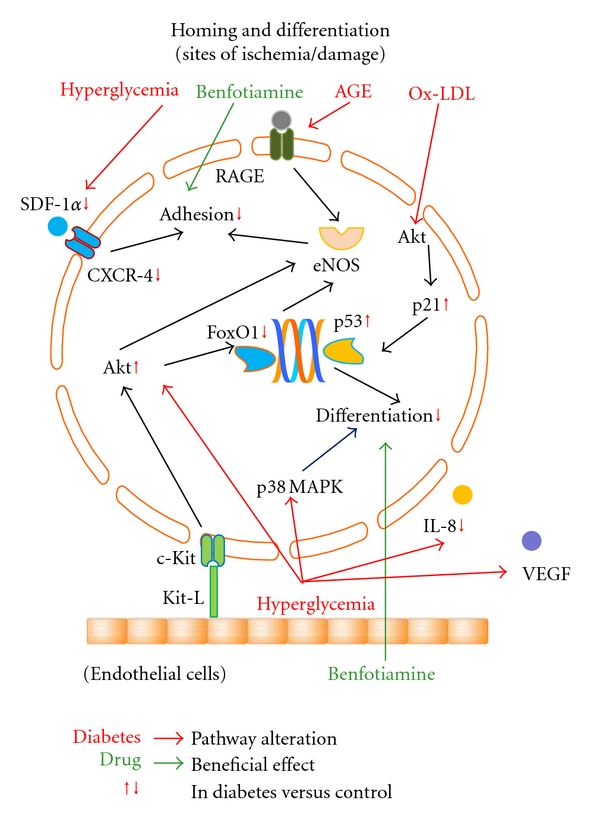
Pathways involved in diabetes-induced EPCs toxicity and possible strategies to reverse EPCs damage during homing. EPCs homing in the sites of ischemia/damage and differentiating into endothelial cells are here represented. Hyperglycemia, Ox-LDL, and AGEs accumulation reduce EPCs adhesion and differentiation ability in both *in vivo* and *in vitro *assays. Benfotiamine, an antioxidant molecule, is able to reverse EPCs dysfunction in homing and differentiation. Diabetes-specific metabolic alterations are in red, linked by red arrows to the pathways they interfere with. Red vertical arrows, next to intracellular or extracellular molecules, indicate that their concentration is diminished or increased in diabetic condition compared to nondiabetic status. Drugs with beneficial effect on EPCs are in green, linked by green arrows to the pathways they interact with. *MMP-9: matrix metalloproteinase-9; ROS: reactive oxygen species; NO: nitric oxide; eNOS: endothelial nitric oxide synthase; SDF-1*α*: stem-cell-derived factor-1*α*; CXCR-4: C-X-C chemokine receptor type 4; Kit-L: c-Kit ligand; Ox-LDL: oxidized low-density lipoprotein; MAPK: mitogen-activated protein kinase; AGEs: advanced glycation end-products; RAGE: receptor for AGE; IL-8: interleukin-8; VEGF: vascular endothelial growth factor. *

To date, several investigators explored a strategy to optimize autologous EPCs function by *ex vivo* conditioning with growth factors/chemoattractants (i.e., SDF-1*α* [21], VEGF [17], IL-8 [13]), antioxidants (i.e., benfotiamine [40]), hormones (i.e., adiponectin [29, 33]), gene therapy (by transfecting EPCs health-relevant genes as eNOS [20], FoxO1 [36], and HIF-1*α* [57]), and clinically available compounds (as p38 MAPK inhibitors [35], CoPP [58], statins [34, 59], and ACE-inhibitors [48, 49]). All these studies showed an improvement in EPCs function, but no application on humans has been tested so far. A detailed description of all molecular mechanisms involved in diabetes-mediated EPCs dysfunction and of the reported compounds potentially able to restore EPCs damage are described in Figures 1, 2, and 3.

### 4.4. Mobilization of EPCs to Overcome EPCs Dysfunction

Dipeptidyl-peptidase-4 (DPP-4) has been recently shown to interfere with EPC function. In a recent clinical trial, Sitagliptin increased the mobilization of EPCs in T2D patients, possibly mediated by SDF-1*α* upregulation [60]. Moreover, EPC mobilization is also induced by physical activity as shown in children exposed to daily exercise [61]. Other strategies, including the induction of EPCs shedding from bone marrow via stem cell mobilizing factors (i.e., GM-CSF), have been shown to be feasible and possibly effective, but it may be argued that a nonspecific cell mobilization would occur and that autologous EPCs are anyway dysfunctional. Unfortunately, EPCs have been demonstrated to be immunogenic [62] and the attractive proposal of transplanting heterologous EPCs pooled from healthy donors would necessarily require the employment of immunosuppressive drugs.

## 5. Conclusions

Functional EPCs represent a prerequisite for a healthy cardiovascular system in diabetic patients. Prevention of diabetes-related macro- and microvascular complications dramatically influences the life expectance and the quality of life of diabetic patients, thus representing a crucial target for physicians. Already available drugs, currently used in clinical practice, and novel compounds should be tested in randomized clinical trials to evaluate their efficacy in normalizing or reverting diabetes-mediated EPCs damage. Moreover, *ex vivo* EPCs expansion, conditioning, and gene therapy might represent potential future strategies to reverse EPCs dysfunction, finally leading to a better cardiovascular outcome.
